# Morphological and Metabolic Features of Brain Aging in Rodents, Ruminants, Carnivores, and Non-Human Primates

**DOI:** 10.3390/ani14192900

**Published:** 2024-10-08

**Authors:** Gianluca Lepore, Sara Succu, Maria Grazia Cappai, Adele Frau, Alice Senes, Marco Zedda, Vittorio Farina, Sergio D. Gadau

**Affiliations:** Department of Veterinary Medicine, University of Sassari, 07100 Sassari, Italy; succus@uniss.it (S.S.); mgcappai@uniss.it (M.G.C.); a.frau4@studenti.uniss.it (A.F.); a.senes1@phd.uniss.it (A.S.); mzedda@uniss.it (M.Z.); vfarina@uniss.it (V.F.); sgadau@uniss.it (S.D.G.)

**Keywords:** brain aging, caloric restriction, mammals, metabolism, neural cells, nutrients

## Abstract

**Simple Summary:**

Brain aging in mammals is characterized by morphological and functional changes in neural cells. This process, although physiological, leads to progressive cerebral tissue volume loss and functional decline, including memory loss motor neuron deficits and behavioral disorders. It is generally accepted that aging is associated with a shift in the proportion between the functional cell (neuron) and support cells (astrocytes) in favor of the latter, which also appear different from those of healthy and young brain tissue. Also, dysfunctional autophagy contributes to altering brain homeostasis. This review summarizes and updates the most recent knowledge about brain aging through a comparative approach, where similarities and differences in some mammalian species are considered.

**Abstract:**

Brain aging in mammals is characterized by morphological and functional changes in neural cells. Macroscopically, this process, leading to progressive cerebral volume loss and functional decline, includes memory and motor neuron deficits, as well as behavioral disorders. Morphologically, brain aging is associated with aged neurons and astrocytes, appearing enlarged and flattened, and expressing enhanced pH-dependent β-galactosidase activity. Multiple mechanisms are considered hallmarks of cellular senescence in vitro, including cell cycle arrest, increased lysosomal activity, telomere shortening, oxidative stress, and DNA damage. The most common markers for senescence identification were identified in (i) proteins implicated in cell cycle arrest, such as p16, p21, and p53, (ii) increased lysosomal mass, and (iii) increased reactive oxygen species (ROS) and senescence-associated secretory phenotype (SASP) expression. Finally, dysfunctional autophagy, a process occurring during aging, contributes to altering brain homeostasis. The brains of mammals can be studied at cellular and subcellular levels to elucidate the mechanisms on the basis of age-related and degenerative disorders. The aim of this review is to summarize and update the most recent knowledge about brain aging through a comparative approach, where similarities and differences in some mammalian species are considered.

## 1. Introduction

Senescence can be described as a physiological process, where cells undergo cell cycle decline while remaining alive [1,2,3]. In general, the senescent processes can provoke both positive and negative effects on tissues and organisms. Positive effects can account for potential tumor suppression [4,5]; for instance, by reducing the frequency of transcription errors in DNA, causing random mutations, by preventing DNA damage, or by promoting DNA repair [6]. Also, wound healing and tissue homeostasis [7,8] could benefit from senescence as a consequence of specific cell factors, such as cytokines and chemokines (SASP) that stimulate the immune system to remove senescent cells [9]. On the other hand, the persistence of senescent cells induces tissue dysfunction, chronic inflammation, and a series of disorders, such as cardiovascular diseases [10], neurological disorders [11], and cancer [12]. The process of senescence elevates the susceptibility to a spectrum of non-communicable diseases, rendering advancing age a primary risk factor for neurodegenerative disorders [13].

**Figure 1:**
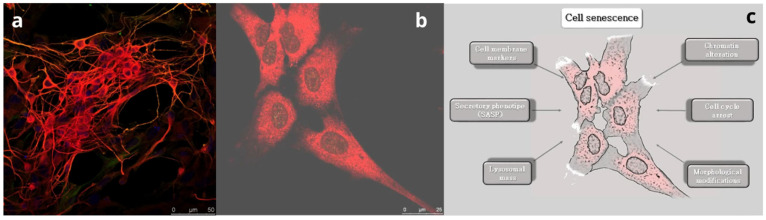
The process of brain aging. (**a**) Sheep fetal healthy neurons positive for the marker β-III tubulin (red). Bar = 50 µm. (**b**) Sheep fetal aged neurons at 30 days of culture. Note the flat and enlarged morphology. Bar = 25 µm. (**c**) Schematic showing the main mechanisms determining cell senescence.

Loss of function induced by aging leads to the modification of neuron components at the subcellular level, which results in compromised function of the whole organ. Macroscopically, this process leads to progressive cerebral tissue volume loss [14] and functional deficits, including memory loss [15,16], reduced motor performance [17], and behavioral disorders [18]. Brain tissue volume modification includes several changes, such as white and gray matter volume loss, cortical thinning, sulcical widening, and ventricular enlargement [19]. Moreover, brain aging is characterized by a slight loss in the number of neuronal cells, as well as a decrease in the diameter and branching density of the dendrites [20,21]. These changes occur in various brain regions, including the hippocampus, where neuronal death can be balanced by a process of neurogenesis [22,23]. Microscopically, brain aging is associated with changes in cell morphology. Indeed, both aged neurons and astrocytes are enlarged and flattened in shape [24,25], and both express pH-dependent β-galactosidase activity. For this reason, the β-galactosidase activity assay has become one of the most commonly used markers of cell aging [26]. Moreover, various mechanisms are considered hallmarks of cellular senescence in vitro (Figure 1).

Among the mechanisms are progressive arrest of the cell cycle, increased lysosomal activity, telomere shortening, oxidative stress, and DNA damage. According to González-Gualda et al. [7], the most common markers for senescence assessment were identified in (i) proteins implicated in cell cycle arrest, such as p16, p21, and p53, (ii) increased lysosomal mass and the corresponding increased levels of senescence-associated β-galactosidase activity, and (iii) increased ROS and SASP expression. This last trait of aging cells was pointed out in rat primary cortical neurons, occurring at an earlier stage than in glial cells, triggered by paracrine activity [27]. Moreover, the same authors found that neurons undergoing senescence developed a senescence-associated secretory phenotype (SASP) that induces paracrine senescence in fibroblasts and chronic inflammation and proliferation of surrounding glial cells. 

One of the most important responses to aging processes is autophagy, which could be referred to as an extreme attempt at cell rescue. Through self-digestion, with the purpose to enhance the turnover of aged intracellular proteins and organelles, the elimination of microorganisms and neoplastic cells also occurs as a side effect of the self-induced anti-aging mechanism [28]. However, dysfunctional autophagy contributes to altering brain homeostasis [27]. Particularly, a study of ours pointed out the expression of the microtubule-associated protein (MAP LC3), involved in autophagic processes, by studying an aging model of primary fetal sheep neurons and astrocytes [25].

Brain senescence processes attract the interest of the scientific community and still represent a hot topic. During the last decades, studies were conducted involving humans, as well as various animal species, such as rodents, ruminants, and carnivores, both in vivo and in vitro. The aim of this review is to summarize and update the most recent knowledge about brain aging through a comparative approach, where similarities and differences across mammalian species are emphasized.

## 2. Assessment of Brain Aging In Vitro

To study the mechanisms of brain aging strictly related to pathological processes, such as Alzheimer’s disease (AD) and Huntington’s disease, analyses are often carried out at cellular levels. A protocol for the extraction and culture of neurons from adult rats and mice is reported in [29]. It produced millions of cortical and hippocampal neurons or neuro-sphere progenitors from each brain. By means of suitable growth factors, regeneration of axons and dendrites in culture allows studies in pharmacology, electrophysiology, development, regeneration, and neurotoxicology. Adult neuro-spheres can be collected as a source of neuro-progenitors and are ethically preferred over embryonic or fetal sources. In addition, senescence is essential for tissue homeostasis, as it limits the proliferative ability of cells. This property was used as a strategy for blocking tumor development [30] in conjunction with apoptosis, which eliminates dysfunctional cells. From this point of view, the mechanisms of cellular senescence were studied in a variety of tumor-derived cells, such as rat neuroblastoma cells [31]. The authors used these cells as an experimental model to study cell senescence [30,32]. Moreover, a murine neuroblastoma cell line was used to test the anti-aging effects of diverse molecules, such as spermidine. This is a molecule that can ameliorate the damage from oxidative stress in aging mice and upregulates the autophagy activity through chromatin acetylation to anti-aging in different cells, from yeasts to fruit flies, and likewise in nematodes and human cells [33,34].

## 3. Astrocytes and Brain Aging

The roles of astrocytes are diverse and essential in brain functioning. They regulate ion homeostasis as well as providing structural support [35] and are key in the establishment and regulation of the blood–brain barrier [36]. In addition, astrocytes provide neurotrophins, such as nerve growth factor (NGF) and brain-derived neurotrophic factor (BDNF), that enhance the function of neurons and oligodendrocytes, promoting survival and differentiation [37,38,39]. Studies also indicated the roles of astrocytes in influencing synaptic function by secreting molecules, such as thrombospondin, glypicans, and cholesterol [40]. They are known to participate in synaptic pruning by releasing signals that induce expression of molecules, such as complement component 1q, in synapses, thereby tagging them for elimination by microglia [41].

Astrocytes are involved in numerous mechanisms related to brain aging. Senescent astrocytes have been detected in the brains of AD patients and AD animal models. In animal models of AD, astrocytes undergo degeneration and atrophy at the early stages of pathological progression, which possibly may alter the homeostatic reserve of the brain and contribute to early cognitive deficits. At later stages of AD, reactive astrocytes are associated with axon plaques, the feature commonly found in animal models and in humans. Changes in morphology and astrocyte reactivity were also observed in rodents [42,43,44]. In animal models of AD, reactive astrogliosis develops in some (e.g., in the hippocampus) but not in all regions of the brain. The essential functions of astrocytes in the adult brain are altered as adults age. Similar to other cell types, aging can cause a loss of normal function in astrocytes, which reduces their ability to properly maintain a healthy CNS environment. This alters their interactions with neighboring cells and contributes to the heightened inflammatory state characteristic of aging [45]. Moreover, the expression of glial fibrillary acidic protein (GFAP) and vimentin increased in mouse astrocytes [46], indicating that such cells become more responsive with age.

## 4. Brain Aging in Rodents

The brains of rodents and, above all, those of rats and mice, have been widely studied to understand the processes of senescence [47]. The quantitative morphological changes in neurons and glia during the aging process were analyzed in the different cortical layers of the frontal cortex of the rats, labeled as I to VI. The parameters analyzed were cortical volume, neuronal density, glial density, and neuronal soma and nucleus areas. No changes with age were found in the volume of the layers, in the neuronal density (except for layer I), or in the area of the neuronal soma. However, older animals showed a 10 to 20% increase in glial density, depending on the layer studied. In addition, there was an age-related decrease in area in the neuronal nucleus in layers II-VI. These results support the idea that the aged frontal cortex undergoes structural changes that may be involved in the morphological basis of memory and cognitive impairments characteristic of aging [48].

## 5. Brain Aging in Ruminants

The brains of sheep have been widely used to study the aging processes [49]. In our laboratories, primary neurons of the sheep brain cortex were firstly setup to highlight age-related morphological features. Morphologically, cell bodies were spindle-like, oval-, or triangular-shaped, with long processes that tended to have contacts with those of the contiguous cells. Cells were identified as neurons by using class III β-tubulin, a marker of neuronal cells. Two morphological types were consistently recognizable: (i) bipolar cells with an oval cell body and (ii) multipolar cells, whose processes formed a wide net with those of adjacent cells. The first work on sheep neurons [50] was developed to evaluate morphological alterations of these cells after oxidative damage, which is widely recognized as a factor that induces senescence. The original protocol was used a second time to assess the expression of topoisomerase βII, a marker of senescence, in fetal sheep neuronal cells. The β isoform of DNA topoisomerase II plays a role in the DNA repair process in non-proliferating cells as neurons, and its expression tends to be downregulated with senescence. The sheep has been chosen here as a suitable experimental model, since it possesses a relatively large brain in comparison to most of the traditional laboratory animals, such as mice or rats. In addition, sheep fetuses allow considerable amounts of brain material to be rapidly collected. Finally, this species is known to be susceptible to severe neurodegenerative diseases, such as Scrapie and Maedi–Visna, in which oxidative stress plays a pivotal role. An imbalance of brain metal homeostasis and associated oxidative stress by redox-active metals, such as iron and copper, has been shown to be involved in several neurodegenerative conditions, including prion disorders [51]. Additionally, in vitro cultures of neural cells from sheep have been previously performed to clarify the mechanisms underlying neuronal ceroid lipofuscinosis (Batten disease). This is widely known as a neurodegenerative age-related disorder occurring both in sheep and in humans, characterized by abnormal fluorescent bodies in many cell types, including neurons [52,53]. In addition, more effective clinical trials to discover the early pathogenic mechanisms of neurodegenerative age-related disorders are needed in large animal models with a complex brain structure (including a more developed cortex with gyri and sulci). From this point of view, the sheep was chosen due to the similarity of its brain structure and size relative to human. Sheep can live for at least 10 years, making them ideal for the study of later-onset diseases, such as the age-related disease AD. In an aging model represented by starvation conditions, sheep neural cells, i.e., neurons and astrocytes, were analyzed by detecting LC3 expression, a marker of autophagy. The results of this study showed that astrocytes have a higher capability than neurons to cope with stress and exhibit a stronger autophagic response [54]. For such reasons, the brain of sheep is a suitable model to clarify the morphological features on the basis of neurodegenerative disorders.

AD involves progressive brain atrophy, neuronal death, synaptic dysfunction, astrogliosis, and the accumulation of protein aggregates in the form of amyloid beta (Aβ) deposits and tau neurofibrillary tangles. Amyloid accumulates as senile plaques and diffuse deposits in the brain around cerebral blood vessels and is termed cerebral amyloid angiopathy (CAA) [55]. The accumulation of Aβ aggregates has not been deeply analyzed in animals; however, it has been reported that AD aggregates are present in the brain of several aged, non-human mammals, including monkeys, bears, dogs, and cheetahs [56].

The cow brain has been used to study the formation of Aβ deposits. In addition, the study of Vallino Costassa et al. [57] demonstrated that Aβ deposits were present in different regions of the cow brain, such as the frontal cortex, cerebellum, and hippocampus. The extracellular deposits were composed of many fine granules frequently associated with microglial cells, as confirmed by double immunofluorescence and Western blot, with an antibody directed to activated microglia. In contrast, reactive astrocytes were only rarely associated with Aβ deposits. In conclusion, the latter study was the first one that fully characterized Aβ deposition in cattle brains. Since the bovine species possesses the same amino acid sequence as humans and displays the same Aβ fragments found in human brains, it provides an interesting model to study Aβ deposition. Aging in general and in bovine species is also associated with a condition called immunosenescence, in which one of the most recognized effects of aging consists in dysregulation of the immune system as a result of defects in both initiation and resolution of immune responses [58]. Immunosenescence is accompanied by alteration of autophagic processes, as viewed in sheep, and a chronic inflammation, termed neuroinflammation. These conditions have been studied extensively and correlated with several neurodegenerative diseases [59,60]. These findings were finally corroborated by the fact that defects in autophagy in cows were associated with various pathological conditions. Those include tumorigenesis, defects in developmental programs, and the build-up of toxic protein aggregates, such as amyloid precursor protein (APP), as well as gliosis and satellitosis involved in neurodegeneration.

Aging in cows is also characterized by an increased concentration of metal ions in the brain that may contribute to a greater increase in free radical production. In their paper, Zatta et al. [61] reported the analysis of Cu, Zn, and Mn in the brain of two series of, respectively, young (8–16 months) and adult (9–12 years) bovines. They found outstanding age-dependent differences in the distribution of Cu and Zn, whose concentrations were markedly higher in older animals. In the presence of transition metals, such as Cu, Mn, and others, superoxide and hydroxyl radicals can be produced. Also, neuroinflammation is an important factor that increases the formation of reactive oxygen species (ROS), which could be a major risk factor in neurodegenerative processes.

## 6. Brain Aging in Carnivores

In the study of brain aging and possible neurological disorders associated with it, one of the most crucial points is the choice of the experimental model. Many animal models are being used, ranging from rodents to non-human primates, each one with advantages but also with associated challenges [62,63,64,65]. For years, the species of choice for this type of study has been rodents, as they have a brain similar in many aspects to that of humans (six-layered cortex) and due to the very easy management and captive breeding. The limitation of these animals, however, is their very short average lifespan and the fact that they do not spontaneously develop some lesions typical of neurological diseases. For example, regarding the study of AD, rodents do not exhibit the same severe gross brain loss in old age as humans, and naturally occurring plaques and tangles have often been difficult to detect, suggesting that they may not exist at all. To replicate the biochemical characteristics of human disease, transgenic models have been employed to overexpress amyloid beta and tau proteins. Other mammals may also be useful to study human neurodegenerative disorders, as they flaunt a more complex anatomy and physiology than rodents. This makes them more comparable to humans in several aspects [66,67]. In recent decades, researchers have paid attention to companion animals, such as dogs [68] and, to a lesser extent, cats [69], as they share the same living environment as humans, and often the same diet, toxins, and stressful stimuli [70,71]. Anatomically, carnivores have a complex brain that resembles that of humans [72,73]. During brain aging, companion animals can show the same anatomical changes as seen in human neurodegenerative disorders, such as AD and its consequent cognitive deficits. As for macroscopic lesions, cerebral and hippocampal cortical atrophy, regional alterations of the prefrontal cortex, and dilatation of the cerebral ventricles were detected in dogs [74]. The atrophy can affect both white and gray matter and is often linked to an imbalance between neuronal maintenance and cell death [75]. Similar anatomical alterations have also been highlighted in elderly cats, where cortical atrophy, enlargement of the cerebral sulci, and an increase in the size of the ventricles have been described [76,77]. These macroscopic alterations are linked to neuronal loss and reduced neurogenesis, as well [78,79]. As for the specific neuronal alterations in neurodegenerative disorders, lesions similar to those observed in AD have been found in elderly dogs and cats. One of the most frequent cellular signs of AD is the appearance and deposition of senile plaques and neurofibrillary tangles in neurons. Plaques contain a toxic peptide called beta-amyloid (Aβ), which derives from the longer Aβ precursor protein (APP) via beta- and gamma-secretase sequential proteolytic cleavage. Aβ forms either extracellular deposits or soluble assembly states. Neurofibrillary tangles are composed of hyperphosphorylated tau protein that fills the cytoplasm of neurons, leading to degeneration. As with most animal models of AD (apart from goats, sheep, and chimpanzees), carnivores may develop Aβ pathology and some tau abnormalities. In terms of plaque composition, carnivores show both commonalities and differences. For example, cats generally form Aβ deposits that are tiny and granular, made up of Aβ1-42 peptides, resembling the early-stage plaques. On the other hand, the majority of human Aβ plaques are bigger and consist of both Aβ1-42 and Aβ1-4071. As for dogs, mature plaques, with their dense Aβ1-40 cores, are less common when compared with humans. Moreover, Aβ1-40 deposits have been shown in cerebral and meningeal blood vessels in both dogs and cats, just as in humans [80,81,82,83]. Because of the cytological and macroscopic alterations in the brain, elderly dogs and cats may show cognitive deficits similar to humans. Aged dogs show deficits in complex learning tasks, including size concept learning, oddity discrimination learning, size discrimination learning, and spatial learning. Further, egocentric spatial learning and memory also decline with age in dogs. Similar to dogs, cognitive alterations in elderly cats have been shown by applying aptitude tests translated from those used on dogs. Cats aged 7–9 years showed both reverse learning and nonmatching in position memory task impairment [77,84].

## 7. Brain Aging in Non-Human Primates

As it is rather difficult to perform research in humans due to numerous ethical and technical considerations, animal models are necessary to recapitulate the complexity of the aging processes. The mouse model is most frequently used for these endeavors; however, it is frequently not the most appropriate model. Non-human primates (NHPs) are important experimental models for studying human conditions because of their close phylogenetic, physiological, and anatomical relationships to humans, their long lifespan [85], and their gyrencephalic brains [86,87,88,89].

Cognitive impairments that accompany aging, even in the absence of neurodegenerative diseases, include deficits in executive function and memory mediated by the prefrontal cortex. Because of the unique differentiation and expansion of the prefrontal cortex in primates, investigations of the neurobiological basis of cognitive aging in non-human primates have been particularly informative about the potential basis for age-related cognitive decline in humans [90]. Frontal and temporal lobe-dependent cognitive functions, including spatial working memory, associative memory, and other executive processes, decline across the human lifespan, even in the absence of neurodegenerative diseases. Non-human primates, such as macaque monkeys, offer several advantages over other animal models in studying healthy cognitive aging. For example, humans and macaques share numerous homologous brain structures and common cytoarchitecture in the prefrontal cortex and temporal lobes, differing mainly in the sizes and laminar thicknesses of the regions [91]. Additionally, similar or identical behavioral tests can be used to assess multiple mental operations in both humans and macaques across their lifespans, allowing for relatively straightforward inter-species comparisons [92]. Importantly, while senescent rhesus macaques do develop some of the pathological markers associated with Alzheimer’s disease, the accumulation is minor, never progressing to meet the criteria for disease diagnosis, which makes them particularly suitable for modeling the normal cognitive aging process [93]. Similar pathological studies have not been carried out on bonnet macaques. Together, these characteristics make macaque models useful for studying the molecular-, cellular-, and systems-level biological correlates of normative age-related changes in executive function and memory [94]. Finally, at the subcellular level, the brain of the macaque has been used to study the morphology changes in astrocytes and microglia in aged subjects [95].

## 8. Aging and Metabolism

Systemic energy demands should be ideally considered as the outcome of the sum of local metabolic activities carried out in different anatomical districts, in view of organ functions, at tissue and specialized cell levels. It is expected that the different roles of specialized cells could display different metabolic extents of draining energy from nutritional substrates per unit of time (in general, during the 24 h) to accomplish very diverse tasks, as a function of specialization in healthy mammals. That way, despite the energy demand at systemic-level estimates of the animal’s overall needs, energy partitioning and conversion at the cellular level should be considered according to cell functions. In addition, the switch of metabolic patterns can also occur, depending on the metabolic level. The metabolic level is conceptually related to the chemical energy stored by the cell and that used to support cell activities. The more active the cell, the higher the expenditure of energy and reload around the 24 h mark. In this way, it is easy to figure out that neurons require an aliquot of energy for maintenance plus extra energy for the extremely high level of activity. However, each time a high metabolic rate is considered, oxidative stress should also be taken into account. The caloric restriction and alternative pathways’ energy utilization and cycle are useful to understand the self-protection strategy put in place by neurons to prevent and limit oxidative damage. With aging, metabolic activities are reduced due to the senescence of the whole body and the progressive decline in the role of the individual (in reproduction, for instance). As aging is a physiological process with progressive tissue deterioration and functional capacity loss [96], the consequent energy requirements should be expected to decline alongside. It is estimated that the energy requirements of the brain drain up to 20–25% of the total energy requirements of the whole individual, despite that the brain mass represents only the 2% of the total body mass in humans [97]. Normal cell metabolism is supported by adequate perfusion, supplying oxygen and glucose as a substrate to allow cell activities. The “membrane pacemaker” theory of aging points to the concept of the optimal combination between high membrane fluidity and low membrane peroxidation, to ensure healthy cell membrane conditions for promoting longevity [98]. It was postulated that the body mass/maximum lifespan in mammals and birds correlates, respectively, directly and inversely with the levels of C18:1n-9 and C22:6n-3 fatty acids in cellular membranes. The physiological basis of such condition may recognize different explanations not fully understood to date. In view of the evidence associated with smaller mammals showing higher metabolic rates, greater fluidity of cell membranes due to extra- and intra-cellular trafficking is expected, though a shorter lifespan is also associated. The fluidity of the cell membrane is tied to the lipid composition and, in particular, to PUFA contents [99]. In view of this, polyunsaturated fatty acid increases the susceptibility to reactive oxygen species (ROS), which are produced at a higher rate as a result of normal metabolic activities. In this way, the smaller body mass and the higher metabolic rate and membrane cell lipid composition require endogenous antioxidant systems to quench the negative effects of ROS, thus being conducive to a higher susceptibility to peroxidation and shorter lifespan, including aging manifestation. To ensure adequate tissue perfusion, endothelial function is, therefore, the necessary condition to support energy provision to cells in each district of the animal body, with the brain as one of the most demanding organs. Against this background, the metabolic approach to brain aging should, therefore, consider adequate perfusion as pivotal, along with cell membrane fluidity and the overall function of gray matter. In general terms, the brain represents the most “fatty” organ, second to proper adipose tissue, in which neutral lipids, such as cholesterol, are the most represented [100]. Also, protein is well represented in healthy human brains, but its decline was observed to progressively enhance along with age [101] due to gray matter reduction. As a matter of fact, as brain fat proportionally increases, inversely to protein on a weight basis, the whole organ is expected to be less and less metabolically active. It is important to establish whether the fat content of the brain has multiple physiological activities [100]. From birth onward, myelination is shown to be a fundamental factor for functional acquisition, and in adulthood the composition of lipids affects the expression of membrane fluidity, stability, and protection. Moreover, the content of lipids on a weight basis changes according to different areas of the brain, showing that the distribution and composition of molecules are also functional in the different activities within the brain regions themselves. In this scenario, it is noteworthy to underline how caloric restriction plays a role in the longevity and activities of the brain. In fact, hormesis, as an expression of an adaptive mechanism to the stress response at the cellular level, has been newly introduced as an endogenous response to caloric restriction in the presence of trans fatty acids of the cell membrane as a trigger to induce mitochondrial biogenesis and oxygen utilization, thus favoring longevity. Such topic deserves a full review, and the reader is invited to refer to the cited literature [101].

In the presence of an oxygen spike thanks to adequate perfusion, aerobic glycolysis is the elective supportive energy pathway for neurons. However, as a buffer mechanism, the astrocytic–neuronal lactate shuttle hypothesis (ANLSH) introduces the lactate as a crucial substrate [102]. In fact, lactate, not pyruvate, is at present acknowledged as the end-product of cerebral aerobic glycolysis, being involved as an energy support molecule to neurons in cases when they need to respond adequately to glutamate excitation. Lactate and NADH production occur under aerobic conditions. Indeed, NADH production follows the conversion of lactate to pyruvate by mitochondrial lactate dehydrogenase, supporting the endogenous antioxidant activity and scavenging ROS [102]. From this perspective, the clever metabolic pathways opportunely adopted by neurons help in the maintenance of membrane stability to promote organ longevity. 

## 9. Conclusions

The study of brain aging is based on the choice of suitable experimental models. Basically, the most used animals were rodents, as they have a brain similar to that of humans and due to the easy management and breeding. In contrast, these animals have very short average lifespans and do not spontaneously develop some lesions typical of neurological diseases. Ruminants could represent a suitable model since they possess a relatively large brain. This allows considerable amounts of brain material to be rapidly collected. Moreover, sheep are known to be susceptible to severe neurodegenerative diseases. Among these are Scrapie and Maedi–Visna, in which oxidative stress plays a pivotal role, and neuronal ceroid lipofuscinosis (Batten disease), occurring in both sheep and humans. Aging in general and specifically in bovine species is also associated with a condition called immunosenescence, a process consisting of the dysregulation of the immune system. This leads to a chronic inflammation termed neuroinflammation, which is correlated with several neurodegenerative diseases. For all these reasons, the brains of ruminants can be studied at cellular and subcellular levels to elucidate the mechanisms on the basis of age-related and degenerative disorders.

Anatomically, unlike rodents, carnivores have a more complex brain that resembles that of humans. During aging, companion animals undergo brain morphological changes, leading to cognitive deficits. One of the most prominent alterations is brain atrophy, which affects several regions and is extended in both white and gray matter. Moreover, senile plaques and neurofibrillary tangles observed in AD can be detected in the brains of elderly dogs and cats. In aged dogs, the brain morphological changes described result in deficits in complex learning tasks, including size concept and spatial learning. For all these reasons, carnivores are suitable for the study of brain aging processes on the basis of cognitive decline.

## Data Availability

Not applicable.
